# Supplementary figures and images for: Characterization of the spontaneous degenerative mitral valve disease in FVB mice
Source: PLoS One. 2021 Sep 2;16(9):e0257022. doi: 10.1371/journal.pone.0257022 (PMC8412250; doi:10.1371/journal.pone.0257022)

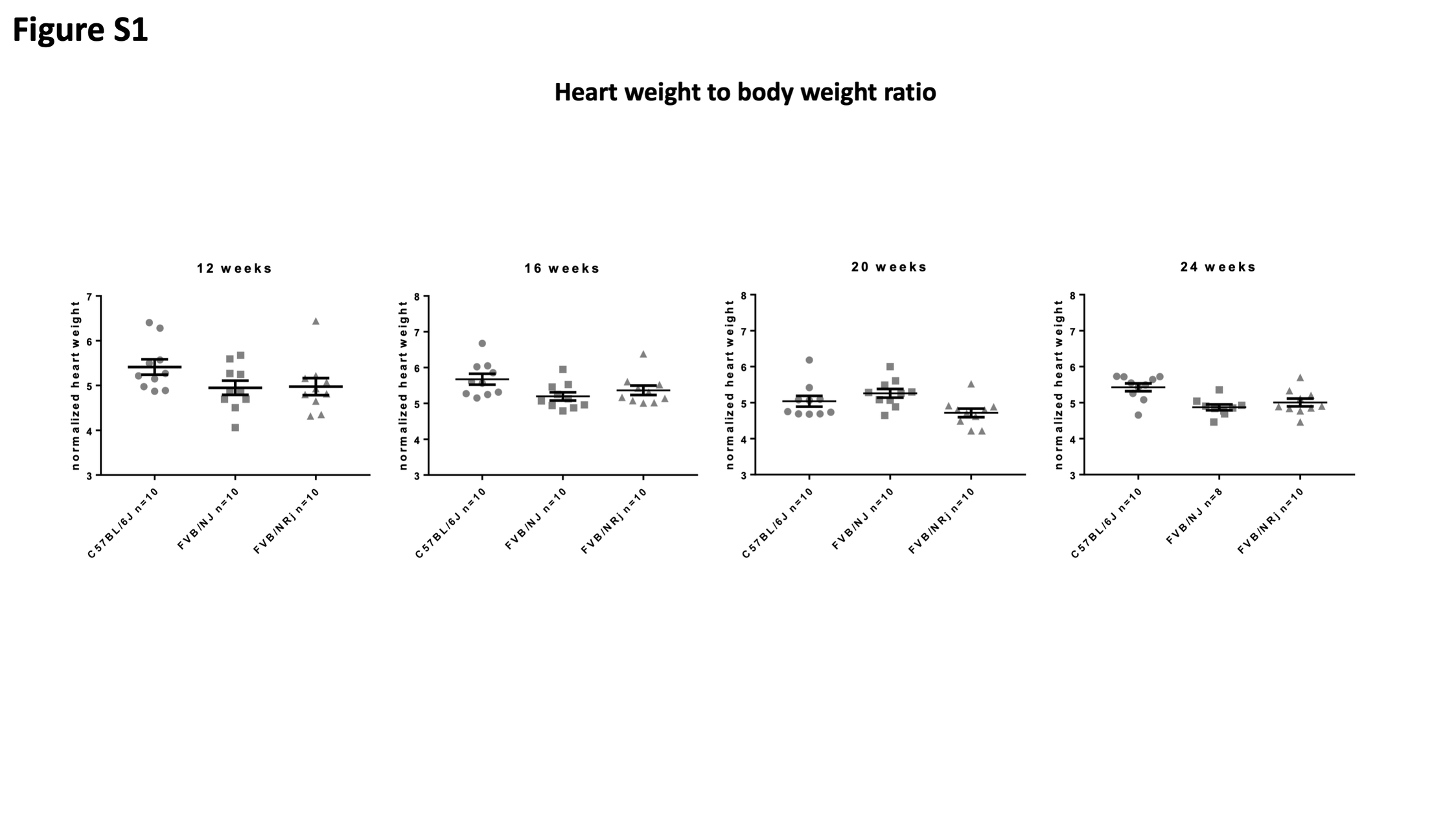

Supplement: S1 Fig — This ratio appreciates myocardial hypertrophy. No significant difference is observed among groups. (TIF) [file pone.0257022.s001.tif]

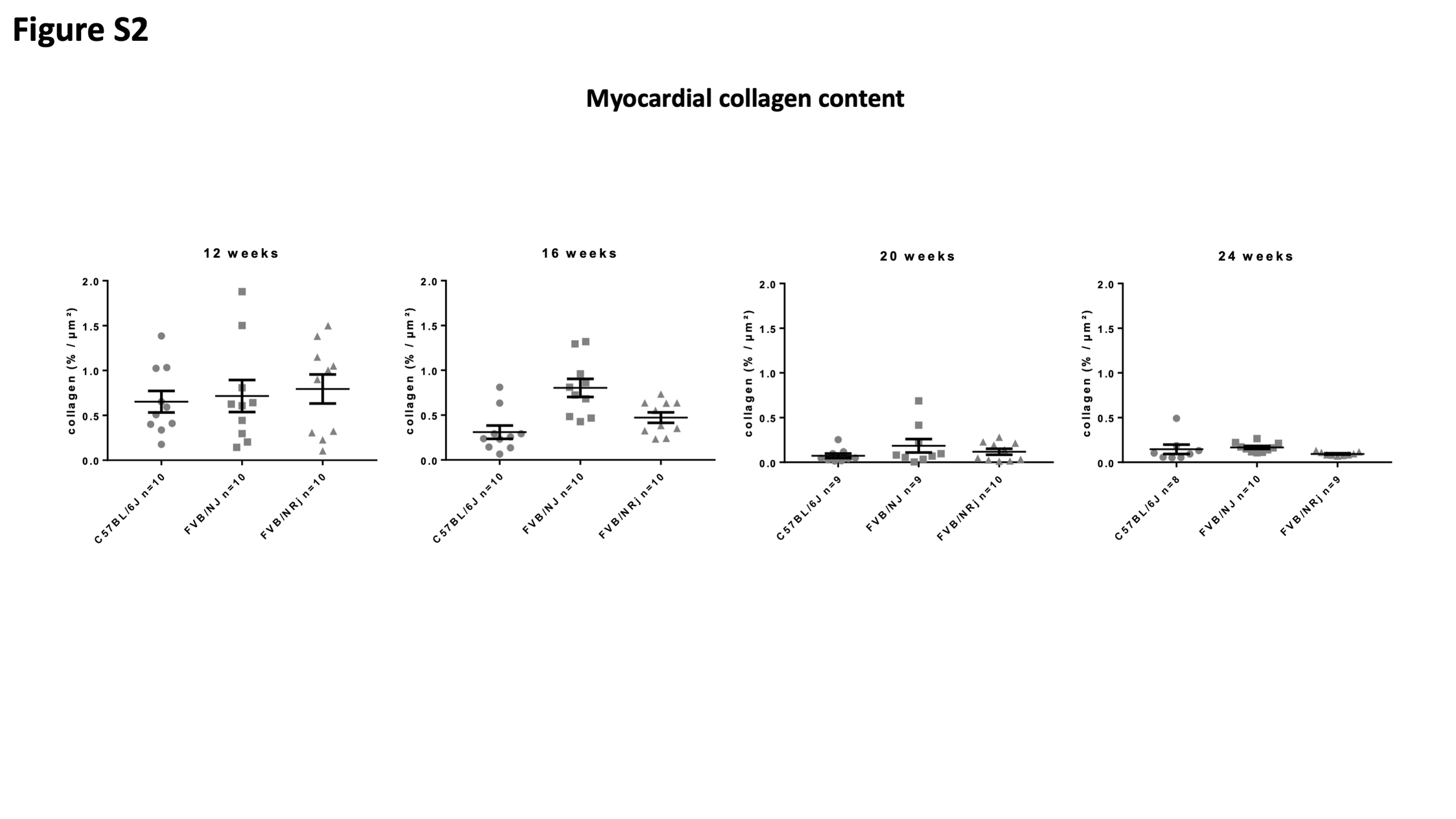

Supplement: S2 Fig — Results are expressed as a percentage of collagen par surface unit. Following cardiac growth, the percentage of collagen decreases because of the physiological cardiac hypertrophy. Nevertheless, no difference was observed between groups at any given time point between 12 and 24 weeks. (TIF) [file pone.0257022.s002.tif]
